# Cryo-EM studies of amyloid-β fibrils from human and murine brains carrying the *Uppsala APP* mutation (Δ690–695)

**DOI:** 10.1186/s40478-025-02120-x

**Published:** 2025-10-03

**Authors:** Mara Zielinski, Fernanda S. Peralta Reyes, Lothar Gremer, Simon Sommerhage, María Pagnon de la Vega, Christine Röder, Thomas V. Heidler, Stina Syvänen, Dieter Willbold, Dag Sehlin, Martin Ingelsson, Gunnar F. Schröder

**Affiliations:** 1 Institute of Biological Information Processing, Structural Biochemistry (IBI-7), Forschungszentrum Jülich, Jülich, Germany; 2 Institut für Physikalische Biologie, Heinrich-Heine University Düsseldorf, Düsseldorf, Germany; 3 Ernst-Ruska-Centre for Microscopy and Spectroscopy with Electrons, Structural Biology (ER-C-3), Forschungszentrum Jülich, Jülich, Germany; 4 Department of Public Health and Caring Sciences, Molecular Geriatrics, Rudbeck Laboratory, Uppsala University, Uppsala, Sweden; 5 Institute of Biological Information Processing, Structural Cell Biology (IBI-6), Forschungszentrum Jülich, Jülich, Germany; 6 Krembil Brain Institute, University Health Network, Toronto, ON Canada; 7 Tanz Centre for Research in Neurodegenerative Diseases, Departments of Medicine and Laboratory Medicine and Pathobiology, University of Toronto, Toronto, ON Canada; 8 Physics Department, Heinrich-Heine University Düsseldorf, Düsseldorf, Germany

## Abstract

Today, 13 intra-amyloid-β (Aβ) amyloid precursor protein (APP) gene mutations are known to cause familial Alzheimer’s disease (AD). Most of them are point mutations causing an increased production or a change in the conformation of Aβ. The *Uppsala APP* mutation (Δ690–695 in APP, Δ19–24 in Aβ) is the first known multi-codon deletion causing autosomal dominant AD. Here, we applied cryo-electron microscopy (cryo-EM) to investigate the structure of Aβ fibrils with the *Uppsala APP* mutation from tg-UppSwe mouse brain tissue. Murine AβUpp(1–42)_Δ19–24_ are made of two identical S-shaped protofilaments with an ordered fibril core of S8-A42. The murine Aβ fold is almost identical to previously described human type II filaments, although the amino acid sequences differ considerably. In addition, we report the cryo-EM structure of Aβ fibrils from the temporal cortex of a patient with the *Uppsala APP* mutation. The observed structure of the human Aβ fold closely resembles previously described type I fibrils. Structural modeling suggests that these fibrils are composed of wild-type Aβ, which implies that AβUpp may be less soluble and thus not readily accessible for cryo-EM image processing and structure determination. Additionally, from the human sample we determined the structures of tau paired helical filaments and tau straight filaments, which are identical to those found in sporadic AD cases. Finally, we present the 3D cryo-EM structures of four dominant AβUpp(1–42)_Δ19–24_ fibril polymorphs, formed in vitro. All four polymorphs differ from the observed folds of Uppsala Aβ in murine and human brain tissue, respectively.

**Supplementary Information:**

The online version contains supplementary material available at 10.1186/s40478-025-02120-x.

## Introduction

Mutations in the amyloid precursor protein (APP) gene (*APP*), presenilin-1 (PS1), and presenilin 2 (PS2) genes (*PSEN1*, *PSEN2*) can lead to early-onset familial Alzheimer’s disease (fAD) characterized by symptom onset before the age of 65 [3]. While *PSEN1* mutations alter ɣ-secretase cleavage and therefore lead to an increase in the amyloid-β (Aβ) 42/40 ratio [8], *APP* mutations affect APP processing and/or Aβ conformation, often leading to increased total Aβ production [15]. Today, more than 50 *APP* mutations are known, of which the majority are pathogenic and 13 of those are located within the Aβ sequence and thus lead to the deposition of mutant Aβ [17]. Most of these intra-Aβ mutations, such as the *Flemish* (A692G, A21G in Aβ), *Arctic* (E693G, E22G in Aβ), *Italian* (E693K, E22K in Aβ), *Dutch* (E693Q, E22Q in Aβ) and *Iowa* (D694N, D23N in Aβ) mutation are single point mutations that result either in an increased production or a change in the conformation of Aβ [4, 7, 12, 17, 19, 21, 24, 36, 37, 41]. The mutation prone E693 residue, which corresponds to residue E22 in Aβ, is involved in both of the known intra-Aβ *APP* deletion mutations, the *Osaka APP* mutation (ΔE693, ΔE22 in Aβ) and the *Uppsala APP* mutation (Δ690-695, Δ19–24 in Aβ). The *Osaka APP* mutation results in a decreased overall production of Aβ with an enhanced generation of toxic Aβ oligomers while fibrillization is inhibited [35]. Solid-state NMR revealed that Aβ40_ΔE22_ aggregates in vitro into fibrils [32] that are significantly different from other previously determined in vitro Aβ fibril structures, whereas the fibrils are surprisingly similar to human wild-type Aβ42 type I filaments mostly found in sporadic AD (sAD) brains [42] and to murine Aβ42_E22G_ fibrils purified from tg-APP_ArcSwe_ mouse brains [44].

**Fig. 1:**
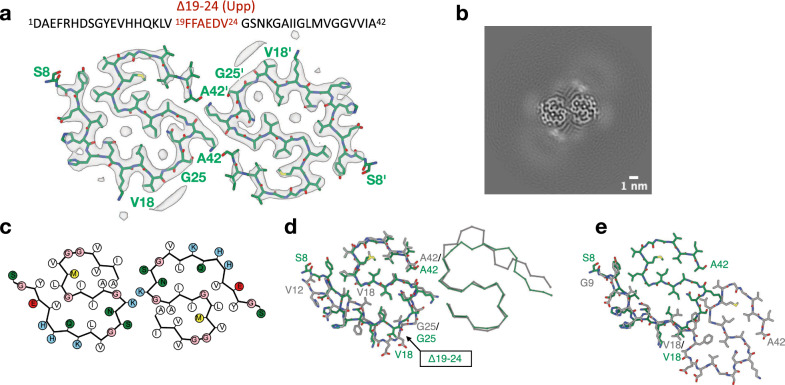
The 3D structure of AβUpp(1–42)_Δ19–24_ purified from tg-UppSwe mouse brain tissue. **a** The cryo-EM density map (in transparent gray) with the atomic model (green). The *Uppsala APP* deletion (Δ19–24) is marked in red within the Aβ42 sequence. **b** Cross-section through the reconstructed fibril density. **c** A schematic of the fold, produced with atom2svg.py [22] (red: acidic; blue: basic; green: hydrophilic; white: hydrophobic; pink: glycine; yellow: sulfur containing). **d** Overlay of the cryo-EM structures of murine Aβ(1–42)_Δ19–24_ fibrils (green) with the cryo-EM structure of human brain-derived type II Aβ42 filaments (gray, PDB 7Q4M). **e** Overlay of the cryo-EM structures of murine Aβ(1–42)_Δ19–24_ protofilaments with the cryo-EM structure of human brain-derived type I Aβ42 filaments (gray, PDB 7Q4B)

The *Uppsala APP* mutation (Fig. 1A) is the first known multi-codon deletion *APP* mutation leading to AD [25]. Discovered in three individuals from a family in Sweden, this mutation gives rise to a dominantly inherited form of early onset fAD. All carriers of the mutation exhibit an early onset of symptoms around the age of 40, coupled with a rapidly progressing disease course. Computed tomography scans display typical AD characteristics, while positron emission tomography (PET) imaging using the amyloid radiotracer ^11^C-labeled Pittsburgh compound B ([^11^C]PiB) reveals only a mildly positive pattern. *Post mortem* analysis of *Uppsala APP* brain tissue is consistent with typical AD pathology, showing abundant deposition of extracellular Aβ plaques, primarily composed of AβUpp(1–42)_Δ19–24_ or an N-terminally truncated form, alongside intracellular tau tangles. Furthermore, the mutation alters APP processing, which results in an increased production of Aβ. In vitro aggregation experiments show a very rapid aggregation of AβUpp(1–42)_Δ19–24_ into amyloid fibrils [25].

Investigation of the molecular mechanisms of AD is often conducted in experimental model systems, such as transgenic mice. We have previously described a murine Aβ fibril structure (murine_Arc_ type I Aβ fibrils from tg-APP_ArcSwe_ mouse) [44] that resembles type I Aβ filaments mostly observed in sAD [42]. We further presented murine Aβ fibril folds (from APP23, ARTE10, tg-APP_Swe_ mice) [44] that are identical to type II filaments predominantly found in fAD and other conditions such as age-related tau astrogliopathy (ARTAG), Parkinson's disease dementia (PDD), dementia with Lewy bodies (DLB), frontotemporal dementia (FTD), and pathological aging (PA) [42]. In addition, the fibril fold found in the brain of APP/PS1 mice (murine type III) [44] shows some similarity to fibrils found in the brain of *Arctic APP* mutation patients [43].

Tg-UppSwe transgenic mice express human APP with the *Swedish APP* mutation (KM670/671NL) and the *Uppsala APP* mutation (Δ690-695) and their brain pathology therefore consists solely of mutant AβUpp(1–42)_Δ19–24_. Like human mutation carriers, tg-UppSwe mice display altered APP processing, which leads to increased production of Aβ that rapidly aggregates into diffuse parenchymal deposits. These Aβ deposits are [^11^C]PiB-PET negative and poorly detected by mAb158 (the murine parent of lecanemab), suggesting that they may be structurally different from Aβ aggregates in other mouse models of Aβ pathology, such as tg-APP_ArcSwe_ [26, 44].

**Fig. 2:**
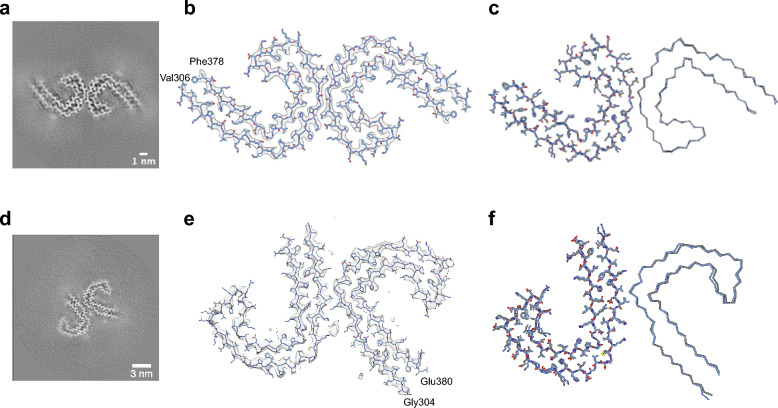
Tau fibrils purified from human brain tissue of an individual with the *Uppsala APP* mutation. **a** Cross-section through the reconstructed fibril density of PHFs. **b** Reconstructed cryo-EM density map of PHFs (gray) and the corresponding atomic model (blue). **c** Comparison of PHFs purified from human *Uppsala APP* mutation brain tissue (blue) and from sAD brain tissue (gray, PDB 5O3L). **d** Cross-section through the reconstructed fibril density of SFs. **e** Overlay of the reconstructed cryo-EM density map of SFs (gray) and the atomic model of SFs from sAD brain tissue (dark blue, PDB 6HRF). **f** Comparison of SFs purified from human *Uppsala APP* mutation brain tissue (blue) and from sAD brain tissue (gray, PDB 6HRF)

**Fig. 3:**
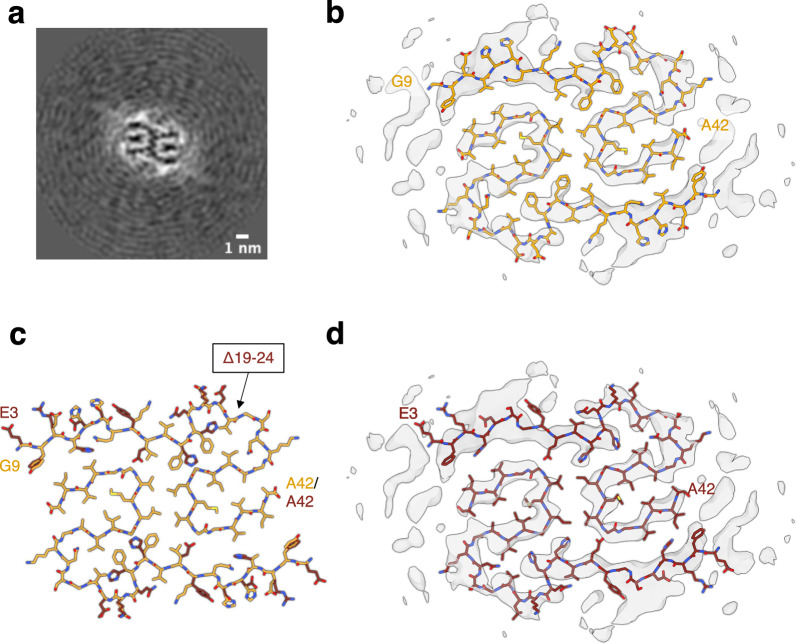
Aβ fibrils extracted from human brain tissue of an individual with the *Uppsala APP* mutation. **a** Cross-section through the reconstructed fibril density. **b** Reconstructed cryo-EM density map (gray) and the atomic model of type I wild-type Aβ42 filaments (orange, PDB 7Q4B). **c** Overlay of the atomic model of type I filaments (orange) and a homology model of AβUpp(1–42)_Δ19–24_ using the atomic model of type I filaments as a template (burgundy). **d** Reconstructed cryo-EM density map (gray) and the fitted homology model of AβUpp(1–42)_Δ19–24_ (burgundy)

**Fig. 4:**
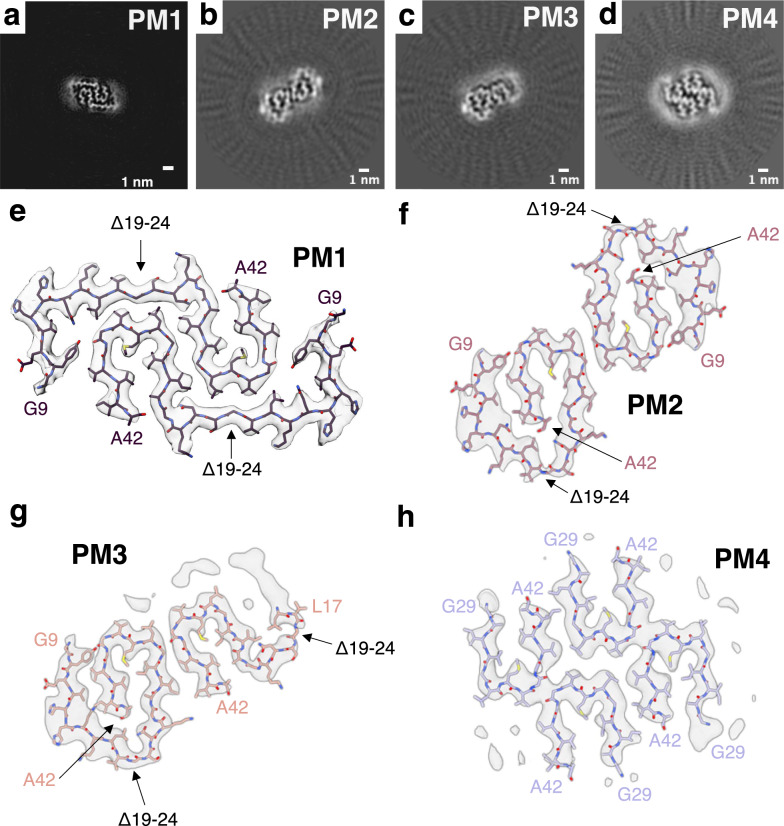
The 3D structure of in vitro AβUpp(1–42)_Δ19–24_. **a**–**d** Cross-sections through the reconstructed fibril densities of **a** PM1, **b** PM2, **c** PM3, and **d** PM4. **e**–**f** The cryo-EM density maps (in transparent gray) of **e** PM1, **f** PM2, **g** PM3, and **h** PM4 with the corresponding atomic models in purple, rose, melon, and lavender, respectively

Here, we determined the cryogenic-electron microscopy (cryo-EM) structure of ex vivo and in vitro AβUpp(1–42)_Δ19–24_ fibrils. We show that AβUpp(1–42)_Δ19–24_ fibrils purified from tg-UppSwe mouse brain tissue, despite the significant six amino acid deletion, show a surprising similarity to type II filaments, which are mostly found in patients with fAD (Fig. 1, Figs. S1A, S2A, D). In addition, we present the structures of tau and Aβ fibrils purified from a patient with the *Uppsala APP* mutation (Figs. 2, 3, S1B,C, S2B,E–G). Finally, in contrast to tg-UppSwe mice, in vitro aggregation of synthetic AβUpp(1–42)_Δ19–24_ peptide results in fibrils that have a high degree of polymorphism and are different from all previously determined Aβ fibril structures (Figs. 4, S2C, H–K).

## Results

### Structure of AβUpp(1–42)Δ19–24 fibrils from tg-UppSwe mouse brain tissue

We determined the cryo-EM structure of AβUpp(1–42)_Δ19–24_ fibrils purified from tg-UppSwe mouse brain tissue to a resolution of 3.2 Å (Fig. 1, Fig. S3A). Murine AβUpp(1–42)_Δ19–24_ fibrils consist of two identical, intertwined S-shaped protofilaments that pack against each other with an approximate pseudo-2_1_ helical symmetry. The ordered core of the fibril, for which atomic model building was possible, extends from residues S8 to A42 (Δ19–24). The S-fold of each protofilament is formed by two hydrophobic clusters around residues (i) Y10, V12, Q15, L17, N27, I 31, and L34, as well as (ii) A30, I32, M35, V40, and A42 (Fig. 1A, C). A hydrogen bond between the carbonyl group at H13 and Q15 in the same monomeric subunit stabilizes the N-terminal arm of the S-fold. The interface between the two protofilaments is formed by salt bridges between K28 and A42' of the adjacent protofilament. As previously observed for other Aβ fibril variants purified from mouse brain tissue [43, 44], murine AβUpp(1–42)_Δ19–24_ fibrils display additional surface-bound densities as well as two smaller, localized densities buried within the protofilament (Fig. 1). The two small densities and the surface-associated density between residues H14 and K16 are in the size range of water molecules or ions. The strong density adjacent to the ε-amino-group of K16, also found in other ex vivo structures [43, 44], suggest the presence of a post-translational modification or a co-factor whose identity remains to be determined.

Weaker micelle-like densities including rod-shaped densities can be observed on the fibril surface in close proximity to residues V18-S26(Δ19–24) and V39-A42. These densities are reminiscent of lipids bound to amyloid fibrils as was observed before [10, 11].

Interestingly, murine AβUpp(1–42)_Δ19–24_ fibrils show a high similarity to human and murine wild-type Aβ42 type II filaments [42, 44], although the sequence register in the N-terminal domain is shifted by six residues (Fig. 1D). Their structures overlap between G25 and A42 with side chain orientations being identical and therefore they share the same overall S-fold as well as exhibit an identical protofilament interface. While murine AβUpp(1–42)_Δ19–24_ fibrils differ from human wild-type Aβ42 type II filaments N-terminally from the mutation site, they are highly similar to the N-terminal domain from G9 to V18 of human type I filaments (Fig. 1E). In both cases the residues E11, H13, H14, K16, and V18 are solvent exposed. In conclusion, the murine AβUpp(1–42)_Δ19–24_ fibril is a hybrid between type I and type II filaments, combining structural elements from both.

### Cryo-EM structures of tau filaments and Aβ fibrils purified from brain tissue of an AD patient with the Uppsala APP mutation

Fibrils were extracted from the temporal cortex of a human individual with the *Uppsala APP* mutation using a previously described sarkosyl extraction method [42] that yields both tau filaments and Aβ fibrils. Immunogold negative stain EM with Nab228 as primary anti-Aβ antibody, which detects the amino-terminal region D1 to E11, showed that the sample contains a majority of unlabeled fibrils with a diameter of ~ 20 Å along with a smaller population of gold-labeled Aβ fibrils mainly observed in larger clumps (Fig. S1B, D). The 3D structure of these unlabeled fibrils was solved using cryo-EM, which reveals that the sample contains a majority of tau fibrils, mainly paired helical filaments (PHFs) alongside some straight filaments (SFs) (see below). Hence, the data confirm the presence of only a small population of Aβ fibrils. This agrees with the fact that AD patients who carry the *Uppsala APP* mutation show pathologically elevated concentrations of tau [25].

We solved the structures of tau PHFs and SFs to a resolution of 3.3 Å and 3.4 Å, respectively (Figs. 2, S3B, C). Atomic model building was possible for the high-resolution reconstruction of PHFs, showing a folded core from residues 306–378 containing all of the R3 and R4 repeats, identical to the previously solved ex vivo polymorph from sAD (PDB ID: 5O3L) [9]. These structures are as well identical to atomic detail, including an extra density in proximity to K317, as identified in a related polymorph at the same K317 position (PDB ID: 6VHL) [2]. The SF density map is in good agreement with the published ex vivo SF structure and adopts the same fold (PDB ID: 5O3T) [9].

It has already been described that mutations in the Aβ sequence may still lead to the same tau PHF fold that can be observed in sAD patients [9, 43]. However, in contrast to patients with the *Arctic APP* mutation, *Uppsala APP* mutation patients also exhibit a minority of SF filaments, whose fold, like that of PHFs, is identical to the SF fold observed in sAD patients [9]. Therefore, the tau pathology is identical to regular sAD even at the molecular level of tau fibrils. However, it is interesting to note that the PHF/SF ratio of 27:1 in the case of the *Uppsala APP* mutation is much higher than the ratio of 2.5:1 observed in the sAD case [9]. This increased ratio fits the cryo-EM analysis of fibrils extracted from brain tissue of a patient with the *Arctic APP* mutation, where no SF filaments were observed [43].

Due to a limited number of available fibril segments the cryo-EM structure of Aβ fibrils was solved to a medium resolution of 5.9 Å, which prevents atomic model building, but reveals the overall fibril fold (Figs. 3, S3D). The reconstructed fibrils consist of two identical, extended S-shaped protofilaments (Fig. 3A) and show the same overall fold as the previously described human wild-type Aβ42 type I filaments [42]. Since the *Uppsala APP* patients express both AβUpp(1–42)_Δ19–24_ and wild-type Aβ(1–42), these fibrils could consist of either peptide. An overlay of the atomic model of wild-type Aβ42 type I filaments with the reconstructed density map provides a good visual fit (Fig. 3B). We have then built a homology model of AβUpp(1–42)_Δ19–24_ based on the wild-type Aβ42 type I filament structure (PDB ID 7Q4B). Interestingly, the homology model fits the reconstructed density map almost equally well (Fig. 3C, D). While the reconstructed map indeed shows some side chain densities at residues F19 and F20 in the wild-type Aβ42 model, the deletion mutation shifts the sequence through the density resulting in the position being occupied by H13 and H14 for the mutated sequence. Similarly, a sidechain density at position Y10 in the wild-type Aβ42 model is occupied by F4 in the mutated sequence. Therefore, at the resolution achieved, the density does not unambiguously determine whether the reconstructed fibrils are composed of wild-type Aβ peptide, mutant Aβ peptide, or a mixture of both.

To address this question, we investigated whether the mutant Aβ sequence could be accommodated by the wild-type Aβ42 type I backbone fold. For this purpose, we performed molecular dynamics (MD) simulations of the AβUpp(1–42)_Δ19–24_ homology model as well as, for comparison, of the wild-type Aβ42 type I fibril for 100 ns each. We observed that the wild-type Aβ structure showed minimal conformation drift (root-mean square deviation (RMSD) of 1.75 ± 0.05 Å during 100 ns). The AβUpp structure instead drifted away from the starting structure within the first 1 ns to an RMSD of 2.58 ± 0.12 Å. We computed a model averaged over the last 10 ns of the 100 ns simulations, which represents the relaxed and equilibrated structure for both AβUpp and wild-type Aβ. These averaged structures of wild-type Aβ and AβUpp were docked into the density map (see Fig. S4). Unlike the wild-type Aβ model, the AβUpp model did not fit well into the density, indicated by the fact that the Cα atoms of the following residues drifted out of the density envelope (defined at the 1-σ threshold): Gly9, Tyr10, Glu11, Leu17, Gly33, and Val36. In particular, the distance between Lys16 to Gly37 in the wild-type Aβ increased by more than 4 Å in the corresponding segment of the AβUpp model (Tyr10 to Gly37), indicating a significant structural deviation. These results indicate that the mutant sequence is not compatible with the atomic model derived from the reconstructed density. We therefore conclude that the fibril structure we have determined from human *Uppsala APP* brain tissue is composed primarily of wild-type Aβ.

### Cryo-EM structures of in vitro AβUpp(1–42)_Δ19–24_ fibrils

In addition, we investigated the structure of AβUpp(1–42)_Δ19–24_ fibrils formed at pH 2 from synthetic AβUpp(1–42)_Δ19–24_ monomers by cryo-EM. Initial screening by negative-stain EM and atomic force microscopy indicated the presence of straight long fibrils with a high degree of polymorphism (Fig. S5). Subsequent cryo-EM analyses revealed the presence of four abundant polymorphs accounting for 43.5% (PM1), 16.9% (PM2), 14.4% (PM3), and 8.4% (PM4) of all fibrils in the dataset. We have earlier described the fold of PM1 and PM2 at low resolution [25]. All polymorphs are different from the ex vivo AβUpp(1–42)_Δ19–24_ structures and different from any other published Aβ fibril structure. However, they all share the same C-terminal U-shape formed by residues K28-A42, which folds around a hydrophobic cluster of residues A30, I32, M35, V40, and A42. This structural element has been observed in many other Aβ(1–42) fibril structures [13, 42]. The 3D structure of the most abundant first polymorph (PM1), that was solved to a resolution of 3.4 Å (Fig. S3E), shows two identical intertwined LU-shaped protofilaments related by a pseudo 2_1_-screw symmetry (Fig. 4A, E). De novo atomic model building was possible for the ordered core between G9-A42(Δ19–24). Residues G9-K28 form the L-shaped domain, preceding the C-terminal U-shaped domain (K28-A42). The N-terminal domain is fixed in its position by a hydrogen bond between Y10 and G37. In addition, the comparably large protofilament interface, which consists of ten residues in each protofilament, is held together primarily by two hydrophobic clusters around residues (i) I32, G33, I31’, and G33’, and (ii) G25, V36’, and L34’, where A’ denotes an amino acid in the respective other protofilament.

PM2 fibrils that were solved to a resolution of 3.9 Å (Fig. S3F), consist of two G-shaped protofilaments that are related by a pseudo 2_1_-screw symmetry (Fig. 4B, F). The atomic model of the ordered core of the fibril between residues G9–A42(Δ19–24) reveals that the inner curve of the G-shape formed by residues K28-A42 folds around the same hydrophobic cluster of residues A30, I32, M35, V40, and A42 that was already observed in PM1 fibrils. The C-terminal U-shaped motif and another hydrophobic cluster, in which the residues G9, Y10, V12, Q15, L17, G37, V39 and I41 are involved, stabilize the G-form of the protofilament. The kink between the inner and the outer G-curve is introduced by a hydrogen bond between N27 and G29. The two G-shaped protofilaments are connected by a hydrophobic interface of residues I31, G33, and L34.The same G-folded protofilament can be observed in the 4 Å reconstruction of PM3 fibrils with the respective other protofilament adopting an S-fold (Figs. 4C, G, S3G). As for PM2 fibrils, atomic model building of the G-shaped protofilament was possible between residues G9-A42 (Δ19–24) while the ordered core of the S-shaped protofilament only spans from L17-A42 (Δ19–24). Hydrophobic interactions between residues I41, V39, and I31 stabilize the interface between the protofilaments.

The cryo-EM structure of PM4 fibrils, solved to a resolution of 3.8 Å (Fig. S3H), reveals that, in contrast to the other three polymorphs observed in vitro, PM4 fibrils are composed of two copies of two identical protofilaments with a high degree of flexibility in the N-terminal domain between residues D1–K28 (Fig. 4D, H). Accordingly, the ordered core of all protofilaments for which atomic model building was possible, extends only from G27–A42. These residues fold into the U-shape as described above. The protofilaments are connected by hydrophobic interactions between (i) G35, and V36, and (ii) I31, G33, L34, V39, and I41.

## Discussion

The *Uppsala APP* mutation leads to a drastic modification to the Aβ peptide and leads to a rare, familial form of AD with an early disease onset. It can be expected that the corresponding Aβ aggregates are different from sporadic and other familial AD cases. We therefore aimed to gain molecular insights into the amyloid forming deposits. Using cryo-EM, we analyzed ex vivo and in vitro AβUpp(1–42)_Δ19–24_ fibrils.

Mouse models of rare familial mutations are valuable because they allow controlled investigation of disease mechanisms initiated by defined genetic changes, enabling causal links between mutation, pathology, and molecular phenotype to be established in vivo. The tg-UppSwe mouse model provides a well-defined system to study the structure of AβUpp(1–42)_Δ19–24_ fibrils that were formed in vivo, because it exclusively produces AβUpp and thereby eliminates any ambiguity from wild-type Aβ. This model proves that AβUpp(1–42)_Δ19–24_ can form well-ordered fibrils in vivo. We have identified a single polymorph in tg-UppSwe mouse brain. The overall fold of this polymorph is almost identical to the type II structure, which has previously been reported in both human brain tissue and other mouse models, where the fibrils are composed of wild-type Aβ42. This finding highlights the exceptional stability and adaptability of the type II fold as it can tolerate a substantial sequence perturbation such as the six-residue deletion.

In addition, there is a striking similarity of the ten N-terminal residues to the type I fibril structure found in humans. However, the N-terminal domain of murine AβUpp(1–42)_Δ19–24_ fibrils is more ordered, with only seven disordered amino acids, whereas wild-type Aβ42 type II filaments have 11 disordered N-terminal residues. A transition between the ordered and disordered part of the chain can be recognized in the cross section of wild-type Aβ42 type II filaments. In contrast, this transition is very abrupt in murine AβUpp(1–42)_Δ19–24_ fibrils: while S8 is still clearly visible in the cross section, D7 is disordered. The observed poor binding affinity of mAb158, the murine parent of lecanemab, to Aβ deposits in tg-UppSwe mice may stem from the inaccessibility of its binding site, spanning amino acids 3–8, as S8 is part of the ordered core in murine AβUpp(1–42)_Δ19–24_ fibrils [26]. In contrast, in murine_Arc_ type I fibrils from tg-APP_ArcSwe_ mice, residues 3–8 are disordered and binding of mAb158 to Aβ deposits can be observed in this mouse model. Similarly, in human type I fibrils, residue 3–8 are also disordered and binding of lecanemab was observed [26, 27, 33, 42, 44]. Several additional densities can be observed in ex vivo murine AβUpp(1–42)_Δ19–24_ fibrils and while their nature remains unknown, it can be assumed that they play a role in the formation of specific fibril folds. Accordingly, the absence of co-factors is one of possibly several reasons why Aβ aggregates in vitro into fibril polymorphs distinct from those found in vivo*.*

In comparison to wild-type Aβ42, recombinant AβUpp(1–42)_Δ19–24_ is much more prone to aggregate into fibrils [25]. The deletion mutation results in a removal of four hydrophobic and two negatively charged residues, which reduces electrostatic repulsion between neighboring monomer layers in comparison to wild-type Aβ42 and possibly promotes aggregation. In human brain tissue of individuals carrying the *Uppsala APP* mutation, the majority of Aβ species is AβUpp(1–42)_Δ19–24_ [25]. However, cryo-EM image processing reveals predominantly wild-type Aβ. This discrepancy suggests that AβUpp(1–42)_Δ19–24_ may aggregate into larger, less soluble clumps that are underrepresented or difficult to visualize by cryo-EM image processing (Fig. S1b, c). It should be noted that if the AβUpp(1–42)_Δ19–24_ fibril structure in humans differs from the fibrils observed in the tg-UppSwe mouse model, this may limit the use of this model in certain translational applications, such as structure-based ligand development, and conclusions about the exact molecular architecture of plaques and their interaction with PET ligands or therapeutics must be drawn with caution.

The in vitro experiments allow us to isolate the effect of the *Uppsala APP* mutation under controlled conditions. By comparing AβUpp(1–42)_Δ19–24_ with wild-type Aβ42 fibrils formed under identical buffer and pH conditions, we can directly assess how this specific sequence deletion influences fibril structure. Interestingly, although the AβUpp variant lacks residues 19–24, the truncated peptide still forms well-ordered fibrils in vitro. This is surprising, given that the deleted residues include part of the central hydrophobic core (KLVFFA), which is frequently described as critical for aggregation. The fact that the fibril formation is preserved suggests that alternative structural stabilization can compensate for this loss, which challenges typical assumptions about essential aggregation motifs. The structural comparison between wild-type Aβ and AβUpp under the same in vitro conditions revealed different polymorphs, as expected given the altered sequence. Nonetheless, the conserved C-terminal turn (residues 30–42) was observed in all AβUpp(1–42)_Δ19–24_ polymorphs, similar to that seen in both in vitro and ex vivo wild-type Aβ42 structures. This highlights a structural constraint or minimal fold element preserved across polymorphs and conditions that is present even in this six-residue deletion variant and which might also contribute to the dominance of the type II fold.

It has been previously reported that PET imaging with the amyloid radiotracer [^11^C]PiB only shows a slightly positive pattern for patients with the *Uppsala APP* mutation despite the presence of abundant Aβ plaques in their brains [25]. This could be explained by structural differences of the surface between AβUpp(1–42)_Δ19–24_ and wild-type Aβ42 fibrils, which possibly results in PiB not recognizing the Uppsala-fold. Therefore, tg-UppSwe mice provide an opportunity to study deposits of human AβUpp in the absence of wild-type Aβ as they only produce the mutated form. Indeed, this mouse model has been shown to be completely [^11^C]PiB negative, which supports the notion of a PiB negative fold of AβUpp fibrils [26]. A similar observation was recently made in the case of the *Arctic* (E22G) APP mutation and the wild-type Aβ fibrils from the APP/PS1 mouse, which both share the same fold and are both negative for [^11^C]PiB [31, 43, 44]. This shared fold structurally differs from human type I and II Aβ42 filaments which are [^11^C]PiB positive. While molecular structure is likely the primary determinant of PiB binding, factors such as aggregation state and packing density may also contribute. To summarize, we solved the fibril structures underlying the tau and Aβ pathologies in a transgenic mouse model and a patient carrying the *Uppsala APP* mutation, the first known multi-codon deletion mutation in the *APP* gene leading to AD [25].

## Methods

### Animals

Generation and characterisation of the transgenic tg-UppSwe mouse model has been described previously [26]. Tg-UppSwe mice carry one copy of the human *APP* gene harbouring the Swedish (*KM670/671NL*) and the Uppsala (*690-695Δ*) *APP* mutations and is maintained through heterozygous breeding on a C57/BL6J-BomTac background. The brain tissue used for the present study was prepared from saline perfused brains of 19–20-months old tg-UppSwe mice (n = 2, female). Breeding and methods for brain isolation were approved by the Uppsala County Animal Ethics boards (5.8.18-20,401/20), following the rules and regulations of the Swedish Animal Welfare Agency, and were in compliance with the European Communities Council Directive of 22 September 2010 (2010/63/EU).

### Human brain tissue

The *Uppsala* APP mutation was identified in two siblings and one cousin, all of whom were evaluated at the Memory Disorder Unit, Uppsala University Hospital. Age at symptom onset was 43 years (sibling 1), 40 years (sibling 2), and 41 years (cousin). At presentation, all three exhibited a manifest cognitive impairment, with Mini-Mental State Examination (MMSE) scores between 20 and 22. Core symptoms included anomia, dyscalculia, apraxia, and visuospatial/executive dysfunction. Sibling 1 developed myoclonus and showed a rapidly progressive course marked by severe anxiety and behavioral dysregulation. Death occurred six years after onset, at age 49. Amyloid fibrils were extracted from post-mortem brain tissue of sibling 1 for structural analysis in this work. The brain weighed 1480 g. On gross examination, ventricular dilatation was observed. Histologically, marked gliosis was present in both limbic and neocortical regions. Widespread tau pathology consistent with Braak stage VI was noted, along with abundant and widespread Aβ plaque deposition corresponding to Thal phase 5. More details are provided in Ref. [25]. The collection and study of the human *APP Upp* brain were approved by the Uppsala Regional Ethical Review Board (2005-103) and The Swedish Ethical Review Authority (2021-04356), respectively.

### Extraction of Aβ fibrils

The extraction of Aβ fibrils from tissue was based on an established sarkosyl extraction procedure [42, 44]. In short, non-fixed frozen human and mouse brain tissue was used for experimentation. 0.46 g of human tissue and two right hemispheres (0.21 g and 0.22 g) from two tg-UppSwe mice were thawed separately and manually homogenized in extraction buffer (20 ml buffer per g original mass; 10 mM Tris–HCl, pH 7.5, 0.8 M NaCl, 10% sucrose, 1 mM EGTA). The homogenization was performed by applying 400 and 300 strokes for the human and the mouse brain tissue, respectively, with similar pressure using a Dounce glass tissue grinder. Further, 10% sarkosyl diluted in H_2_O (Sigma-Aldrich) was added to each homogenate and was thoroughly mixed 30 times by pipetting up and down, reaching a sarkosyl concentration of 2%.

The homogenates were incubated for 1 h at 37 °C in a thermoblock and centrifuged at 10,000×*g* for 10 min at 4 °C. Subsequently, the supernatants were ultracentrifuged at 100,000×*g* for 60 min at 4 °C (Beckman Coulter Optima MAX-XP, TLA55 fixed-angle rotor). The resulting supernatants were discarded and extraction buffer (1 ml g^–1^ original tissue mass) was added and mixed with the pellets, followed by 5000×*g* centrifugation for 5 min at 4 °C. Then, the supernatants were threefold diluted in dilution buffer (50 mM Tris–HCl, pH 7.5, 0.15 M NaCl, 10% sucrose, 0.2% sarkosyl) and ultracentrifuged at 100,000×*g* for 30 min at 4 °C. After the supernatants were discarded, resuspension buffer (20 mM Tris–HCl, pH 7.4, 50 mM NaCl) was added (100 µl g^–1^ original tissue mass) to the insoluble Aβ fibril-rich final pellets from mouse and human tissue. One of the mouse tissue derived pellets was used for negative staining and immunogold labelling, the second pellet was used for cryo-EM analysis, whereas the same human final pellet was used for all the imaging and cryo-EM analysis.

The tau SF fibril structures were determined from a second fibril extraction attempt. The overall procedure was based on the protocol by Hoq et al. [18]. Briefly, 0.44 g of sporadic AD human tissue was thawed and manually homogenized in extraction buffer consisting of 10 mM Tris–HCl, pH 7.4, 0.8 M NaCl, 1 mM EGTA, 5 mM EDTA, and 10% sucrose with one complete mini Roche protease inhibitor tablet dissolved in 200 mL buffer. Samples were centrifuged at 16,000×*g* for 20 min at 4 °C. The resulting supernatants were brought to 1% sarkosyl final concentration. Supernatants were incubated at 25 °C while shaking at 300 rpm. After centrifugation at 100,000×*g* for 1 h at 4 °C, the sarkosyl-insoluble pellets were resuspended in 10 μl/g tissue 50 mM Tris–HCl, pH 7.4. The pellet was diluted in extraction buffer and centrifugated at 16,000×*g* for 30 min at 4 °C. The supernatant was centrifuged at 100,000×*g* for 1 h at 4 °C and the final pellet resuspended in 20 mM Tris–HCl, pH 7.4, with 100 mM NaCl. The final sample was frozen in liquid nitrogen and stored at − 80 °C until further use.

### Sample preparation of the in vitro sample

Synthetic AβUpp(1–42)_Δ19–24_ was purchased (Innovagen AB). The peptides were prepared under the same conditions as previously described [13] and incubated in 30% (v/v) acetonitrile (AcN), 0.1% (v/v) trifluoroacetic acid (TFA) at pH 2 (~ 300 µM monomer concentration). Monomer to fibril conversion occurred over several weeks at room temperature under quiescent conditions.

### Atomic force microscopy of the in vitro sample

For Atomic Force Microscopy imaging, the sample solution was diluted 1:30 in 30% (v/v) AcN, 0.1% (v/v) TFA in water. Afterwards, 5 µL of the diluted sample solution was applied to a freshly cleaved muscovite mica and dried with a stream of N_2_ gas. Imaging was performed in intermittent contact mode (AC mode) in a Nano Wizard 3 atomic force microscope (JPK, Berlin) using a silicon cantilever (OMCL-AC160TS, Olympus) with a typical tip radius of $9 \pm 2\,{\text{nm}}$. The images were processed using Gwyddion (version 2.61) [23].

### Negative stain electron microscopy

#### Murine

2 µL of the final sarkosyl insoluble fraction was applied onto a glow-discharged 300 mesh carbon-coated copper grid (EM Sciences, ECF300-Cu). The sample was incubated for 2 min, excess liquid was blotted off with filter paper and the grid was washed once with H2O. 2µL of 1% (w/v) uranyl acetate (UrAc) were applied on the top of the grid, following a 1 min incubation. The UrAc was removed with filter paper and the grid was air-dried.

#### In vitro

3 µL of the synthetic sample were applied onto a glow-discharged 300 mesh carbon-coated copper grid (EM Sciences, ECF300-Cu). The sample was incubated on the grid for 2 min and excess liquid was blotted off with filter paper. 3 µL of 2% (w/v) UrAc were applied onto the grid and incubated for 1 min. The UrAc was removed with filter paper and the grid was air-dried.

TEM images were acquired using a ThermoFisher Scientific Talos 120C at an acceleration voltage of 120 kV. Images were collected on a 4 k × 4 k Ceta 16M CEMOS camera using Thermo Scientific Velox Software.

### Immunogold negative stain electron microscopy of ex vivo samples

Immunogold negative-stain grids for electron microscopy were prepared following a published protocol [14, 27, 44]. For the murine and the human sample, 2 µL and 3 µL sample were applied onto glow-discharged 300 mesh carbon-coated copper grid (EM Sciences, ECF300-Cu), respectively. After 2 min incubation, the sample was washed once with H_2_O, placed in blocking buffer (99 mL PBS, pH 7.4, 100 μL Tween-20, 1 mL 30% IgG-free bovine serum albumin) for 15 min, following incubation with Nab228 (Sigma-Aldrich) primary antibody at a concentration of 2 µg/mL for 1–2 h. The grid was washed with washing buffer (100 mL PBS, pH 7.4, 100 μL Tween-20, 100 μL 30% IgG-free bovine serum albumin) and then incubated with a 6 nm gold-conjugated anti-mouse secondary antibody (diluted 1:20 in blocking buffer, Abcam) for 1 h. Afterwards, the grid was washed with washing buffer and H_2_O before staining with a 1% (w/v) UrAc solution for 1 min. The grids were air-dried, and EM images were acquired as described above. Representative images are shown in Fig. S1.

### Cryo-EM image acquisition and data preprocessing

For cryo-EM imaging of the murine sample, 2 µL of Aβ fibril sample from a single tg-UppSwe mouse brain was applied to holey carbon grids (Quantifoil 1.2/1.3, 300 mesh), blotted with filter paper for 6 s and plunge frozen in liquid ethane using a ThermoFisher Scientific Vitrobot Mark IV, set at 95% humidity and 4°C. Data acquisition was performed on a ThermoFisher Scientific Titan Krios G4 operating at 300 kV using a Falcon IV detector in counting mode.

Prior to cryo-EM imaging of the human sample, the solution was centrifuged at 5000×*g* for 6 min at 4°C, the supernatant was removed, and the remaining pellet was resuspended in resuspension buffer. The sample was vortexed for 2 s and centrifuged at 5000×*g* for 6 min at 4 °C. Afterwards, the sample was transferred to a 500 µL LoBind Eppendorf tube and sonicated in pulses of 3 min (10 s on, 20 s off) in an ultrasonic water bath. Then, 2 µL of the Aβ fibril sample was applied to holey carbon grids (Quantifoil 1.2/1.3, 300 mesh), blotted with filter paper for 6 s and plunge frozen in liquid ethane using a ThermoFisher Scientific Vitrobot Mark IV, set at 95% humidity and 4°C. Data acquisition was performed on a ThermoFisher Scientific Titan Krios G4 operating at 300 kV using a Falcon IV detector in counting mode.

For cryo-EM imaging of the in vitro sample, 2 µL sample solution was applied to holey carbon grids (Quantifoil 1.2/1.3, 300 mesh), blotted with filter paper for 5 s and plunge frozen in liquid ethane using a ThermoFisher Scientific Vitrobot Mark IV, set at 95% humidity and 4 °C. Data acquisition was performed on a ThermoFisher Scientific Talos Arctica operating at 200 kV using a Gatan Bioquantum K3 direct electron detector in counting mode with a Gatan Bioquantum energy filter with a slit width of 20 eV. For all grids automated collection was directed by EPU data collection software. Further details are given in Table S1. For helical reconstruction of all datasets, gain-corrected movie frames were aligned and summed into single micrographs on-the-fly using Warp [34]. CTF estimation was performed using CTFFIND4.1 [29].

A second dataset was collected from the second fibril extraction, from which the tau SF structure was determined. The data collection protocol was the same as described above for the first dataset; details are given in Table S1.

### Helical reconstruction

Helical reconstruction was performed using the helical reconstruction methods in RELION [16, 45]. The helical image processing follows the procedures described by Scheres (ref. [30]).

For the cryo-EM dataset of Aβ fibrils purified from tg-UppSwe mouse brain tissue, fibrils were picked automatically using crYOLO [38, 39]. Reference-free 2D classification at a box size of 800 pixel, downscaled to 200 pixel, was performed to get an overview on polymorph distribution and to discard false positives from autopicking as well as lower quality fibril segments. Afterwards, fibril segments were re-extracted at 300 pixel box size and the original pixel size of 0.808 Å/pixel. Further reference-free 2D classification was performed to discard lower quality fibril segments. A featureless cylinder with a diameter of 140 Å was lowpass-filtered to 40 Å and used as initial 3D reference. Iterative 3D classification and 3D refinement with refinement of the helical parameters was performed to yield a higher resolution reconstruction. 3D auto-refinement and subsequent post-processing was performed to compute the final map and to calculate the resolution according to gold-standard Fourier Shell Correlations at 0.143 applying a soft-edged solvent mask. Additional information can be found in Table S1.

For the cryo-EM dataset of fibrils purified from brain tissue of an AD patient with the Uppsala mutation, fibrils were picked manually. Fibril segments were extracted at a box size of 1200 pixel, downscaled to 300 pixel. Reference-free 2D classification was performed to separate different fibril types. The particle set was split into subsets that were processed individually. Particles of all subsets were re-extracted at an image processing box size of 256, 270, or 300 pixel. For PHF tau filaments, SF tau filaments, and Aβ fibrils, an initial 3D reference was computed de novo from multiple 2D class averages assuming a helical rise of 4.75 Å and a twist value calculated from the crossover-distance of each fibril observed from the larger box 2D class averages using the *relion_helix_inimodel2d* command [30]. The initial 3D references were low-pass filtered to 6 – 8 Å depending on their quality. Iterative 3D classification and 3D refinement with refinement of the helical parameters was performed to yield a higher resolution reconstruction. 3D auto-refinement and subsequent post-processing was performed to compute the final map and to calculate the resolution according to gold-standard Fourier Shell Correlations at 0.143 applying a soft-edged solvent mask. Additional information can be found in Table S1.

For the cryo-EM dataset of in vitro AβUpp(1–42)_Δ19–24_ fibrils, fibrils were picked manually. Fibril segments were extracted at a box size of 810 pixel, downscaled to 270 pixel. Reference-free 2D classification was performed to separate different fibril types. The particle set was split into subsets that were processed individually. Particles of all subsets were re-extracted at an image processing box size of 270 pixel. For PM1 and PM4 fibrils, an initial 3D reference was computed de novo from one large 2D class average assuming a helical rise of 4.75 Å and a twist value calculated from the crossover-distance of each fibril observed from the larger box 2D class averages using the *relion_helix_inimodel2d* command. For PM2 and PM3 fibrils, a featureless cylinder with a diameter of 64 Å was used as initial 3D reference. Cylinders were initially low-pass filtered to 40 Å, reconstructed de novo initial models were low-pass filtered to 8 Å. Iterative 3D classification and 3D refinement with refinement of the helical parameters was performed to yield a higher resolution reconstruction of all polymorphs. For PM1, particle images with a CTF fit resolution worse than 3.5 Å were excluded from processing. 3D auto-refinement and subsequent post-processing was performed to compute the final map and to calculate the resolution according to gold-standard Fourier Shell Correlations at 0.143 applying a soft-edged solvent mask. Additional information can be found in Table S1.

#### Handedness

For the tg-UppSwe mouse fibril structure, obtained resolution of 3.2 Å was sufficient to determine that the fibril has a left-handed helical twist, as the central density region was sufficiently well-resolved to reveal the characteristic bumps associated with the backbone carbonyl oxygen atoms. For the human Aβ structure the handedness was derived from the published type-1 structure (PDB ID 7Q4B). The PHF and SF tau fibril structures are identical to known polymorphs (see below) with known handedness, both have a left-handed twist. For the in vitro structures, we determined the handedness by comparison with the entire C-terminal U-shaped motif, which is known from several high-resolution Aβ fibril structures. We have compared the C-terminal motif (residues 30–40) from PDB ID 7Q4B with both the left-handed and right-handed fibril density (cf. Fig. S6) and found clearly better fit of this motif to the left-handed fibrils for all four polymorphs.

### Model building and refinement

For murine AβUpp(1–42)_Δ19–24_ fibrils an atomic model was built de novo into the density map and refined using an iterative procedure of manual modeling in COOT [5] and automated refinement in PHENIX [1]. Side chain rotamers were refined manually monitoring Ramachandran outliers and clash scores using MolProbity [40].

For the human sample, atomic model building was possible for the high-resolution reconstruction of PHF tau filaments. Here, a previously determined PHF structure (ref. [9], PDB code: 5O3L) was fitted into the reconstructed map using ChimeraX [28] and used as initial model. This model was refined into the density map using an iterative procedure of manual modeling in COOT and automated refinement in PHENIX. Side chain rotamers were refined manually monitoring Ramachandran outliers and clash scores using MolProbity. The SF density map fitted perfectly to the previously published model PDB ID 5O3T [9].

For all four in vitro polymorphs, atomic models were built de novo into the computed cryo-EM reconstructions using COOT. Side chain rotamers were refined manually monitoring Ramachandran outliers and clash scores using MolProbity. All models were afterwards refined using an iterative procedure of manual modeling in COOT and automated refinement in PHENIX.

In all cases, 5 layers of the fibril model were built and NCS restraints between all chains were using during the refinement in PHENIX. Final refinement was performed with ISOLDE [6]. ChimeraX was used for molecular graphics and analyses. Additional information on all atomic models can be found in Table S1.

### MD simulations of wild-type and and AβUpp(1–42)_Δ19–24_ atomic models

The simulation of the wild-type Aβ(1–42) structure was based on the atomic model with PDB ID 7Q4B [42]. A homology model of AβUpp(1–42)_Δ19–24_ was built using the wild-type structure PDB ID 7Q4B as a template. All side chain rotamers were chosen to be as close as possible to the template structure. For each model, a fibril fragment consisting of 10 layers, i.e., 20 peptide chains, were built. Water molecules and ions (sodium and chloride) were added at a concentration of 150 mM. In the case of the wild-type structure, the cations in the PDB model close to Glu22 and Asp23 were retained and modeled as Na^+^ ions. The GROMACS software (version 2019.3) [20] was used to perform molecular dynamics simulations with the Amber99SB-ILDN force field and the TIP3P water model. Equilibration at 300 K was carried out for 1 ns with position restraints on the Cα atoms to relax the solvent and protein structure. Afterwards, 100 ns production runs without any restraints were performed for each model.

## Supplementary Information

**Additional file 1:** Supplementary Figures.

**Additional file 2:** Supplementary Table S1.

## Data Availability

Cryo-EM maps have been deposited to the Electron Microscopy Data Bank (EMDB) and to the Protein Data Bank (PDB) under the following accession numbers: EMD-50436 and 9FH1 (murine AβUpp(1-42)Δ19–24); EMD-50437 and 9FH2 (in vitro polymorph 1); EMD-50438 and 9FH3 (in vitro polymorph 2); EMD-50439 and 9FH4 (in vitro polymorph 3); EMD-50440 and 9FH5 (in vitro polymorph 4); EMD-50441 and 9FH6 (human ex vivo tau paired-helical filament); EMD-50442 (human ex vivo amyloid-beta fibril).
