# Supplementary material for: Cryo-EM studies of amyloid-β fibrils from human and murine brains carrying the Uppsala APP mutation (Δ690–695)
Source: Acta Neuropathol Commun. 2025 Oct 3;13:209. doi: 10.1186/s40478-025-02120-x (PMC12492897; doi:10.1186/s40478-025-02120-x)

Cryo-EM studies of amyloid-β fibrils from human and murine brains carrying the *Uppsala APP* mutation (Δ690–695)

SUPPLEMENTARY DATA

Mara Zielinski[1], Fernanda S. Peralta Reyes[2], Lothar Gremer[1,2*], Simon Sommerhage[1,3], María Pagnon de la Vega[4], Christine Röder[1,2], Thomas V. Heidler[3,5], Stina Syvänen[4], Dieter Willbold[1,2], Dag Sehlin[4], Martin Ingelsson[4,6,7*], Gunnar F. Schröder[1,3,8*]

[1]Institute of Biological Information Processing, Structural Biochemistry (IBI-7), Forschungszentrum Jülich, Jülich, Germany

[2]Institut für Physikalische Biologie, Heinrich-Heine University Düsseldorf, Düsseldorf, Germany

[3]Ernst-Ruska-Centre for Microscopy and Spectroscopy with Electrons, Structural Biology (ER-C-3), Forschungszentrum Jülich, Jülich, Germany

[4]Department of Public Health and Caring Sciences, Molecular Geriatrics, Rudbeck Laboratory, Uppsala University, Uppsala, Sweden

[5]Institute of Biological Information Processing, Structural Cell Biology (IBI-6), Forschungszentrum Jülich, Jülich, Germany

[6]Krembil Brain Institute, University Health Network, Toronto, Ontario, Canada

[7]Tanz Centre for Research in Neurodegenerative Diseases, Departments of Medicine and Laboratory Medicine & Pathobiology, University of Toronto, Toronto, Ontario, Canada

[8]Physics Department, Heinrich-Heine University Düsseldorf, Düsseldorf, Germany.

This file includes:

Supplementary Figures S1-S8

Supplementary Table 1

Supplementary Figures

Figure S1: Immunogold negative-stain electron microscopy images of the purified Aβ and tau fibrils from (**a**) tg-UppSwe mouse brain tissue, and (**b**, **c**) human brain tissue of an AD patient with the Uppsala mutation using Nab228 as primary antibody detecting the N-terminal region D1-E11.

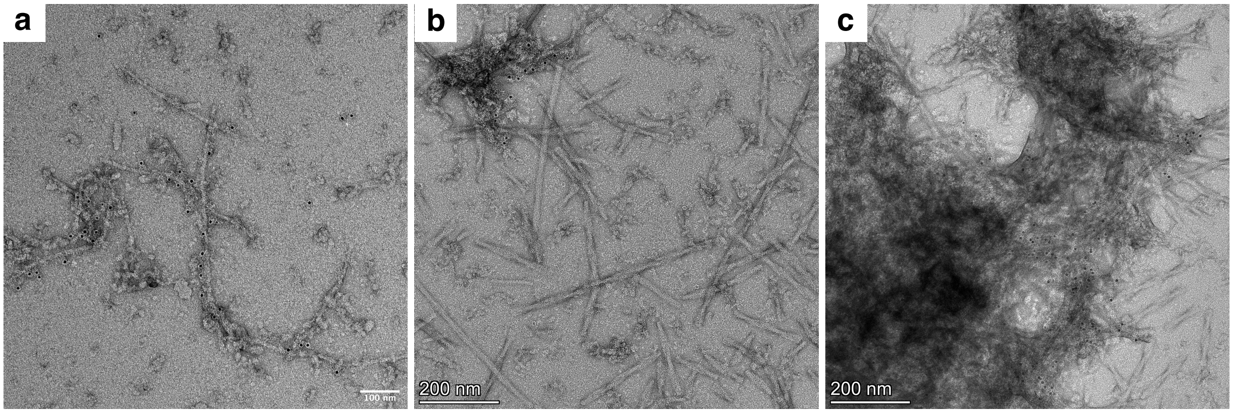

Figure S2: Cryo-EM micrographs and density maps. One exemplary cryo-EM micrograph of (**a**) the *ex vivo* tg-UppSwe sample, (**b**) the *ex vivo* human brain sample, and (**c**) the *in vitro* sample. 3D reconstructions of (**d**) AβUpp(1-42)$_{\Delta 19\text{-}24}$ fibrils purified from tg-UppSwe mouse brain tissue, (**e**) Tau PHFs purified from human brain tissue of an individual with the Uppsala mutation, (**f**) Tau SFs purified from human brain tissue of an individual with the Uppsala mutation, (**g**) Aβ fibrils purified from human brain tissue of an individual with the Uppsala mutation, (**h**) *in vitro* AβUpp(1-42)$_{\Delta 19\text{-}24}$ PM1 fibrils, (**i**) *in vitro* AβUpp(1-42)$_{\Delta 19\text{-}24}$ PM2 fibrils, (**j**) *in vitro* AβUpp(1-42)$_{\Delta 19\text{-}24}$ PM3 fibrils, and (**k**) *in vitro* AβUpp(1-42)$_{\Delta 19\text{-}24}$ PM4 fibrils.

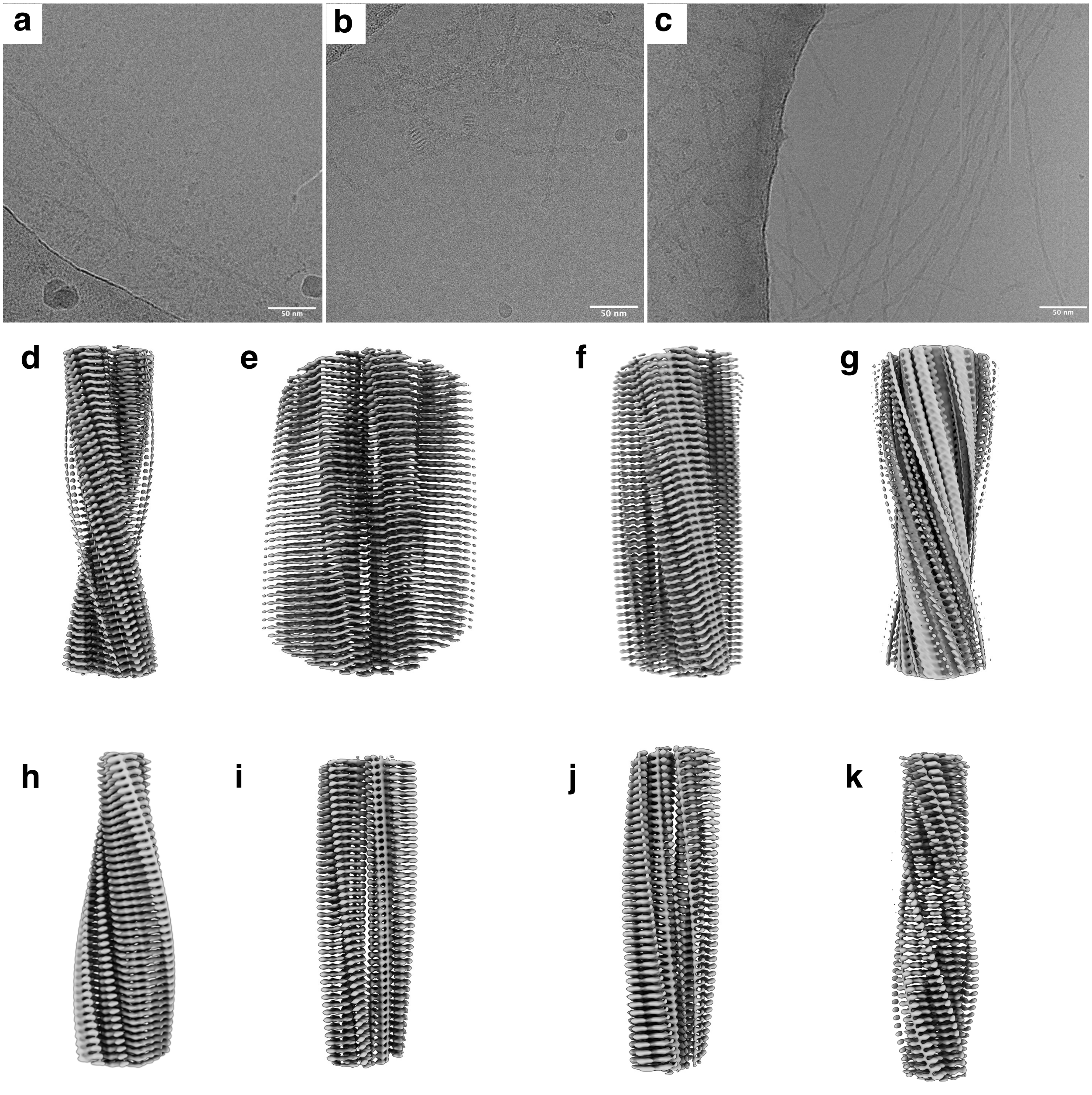

Figure S3: FSC curves for cryo-EM maps of (**a**) AβUpp(1-42)$_{\Delta 19\text{-}24}$ purified from tg-UppSwe mouse brain tissue, (**b**) PHF tau filaments purified from human brain tissue of an individual with the Uppsala mutation, (**c**) SF tau filaments purified from human brain tissue of an individual with the Uppsala mutation, (**d**) Aβ fibrils purified from human brain tissue of an individual with the Uppsala mutation, (**e**) *in vitro* AβUpp(1-42)$_{\Delta 19\text{-}24}$ PM1, (**f**) *in vitro* AβUpp(1-42)$_{\Delta 19\text{-}24}$ PM2, (**g**) *in vitro* AβUpp(1-42)$_{\Delta 19\text{-}24}$ PM3, and (**h**) *in vitro* AβUpp(1-42)$_{\Delta 19\text{-}24}$ PM4. In each panel, the solid blue curve shows the FSC between two independently refined half-maps (even versus odd), and the dashed green curve shows the FSC between the final reconstructed map and the corresponding atomic model–derived map.

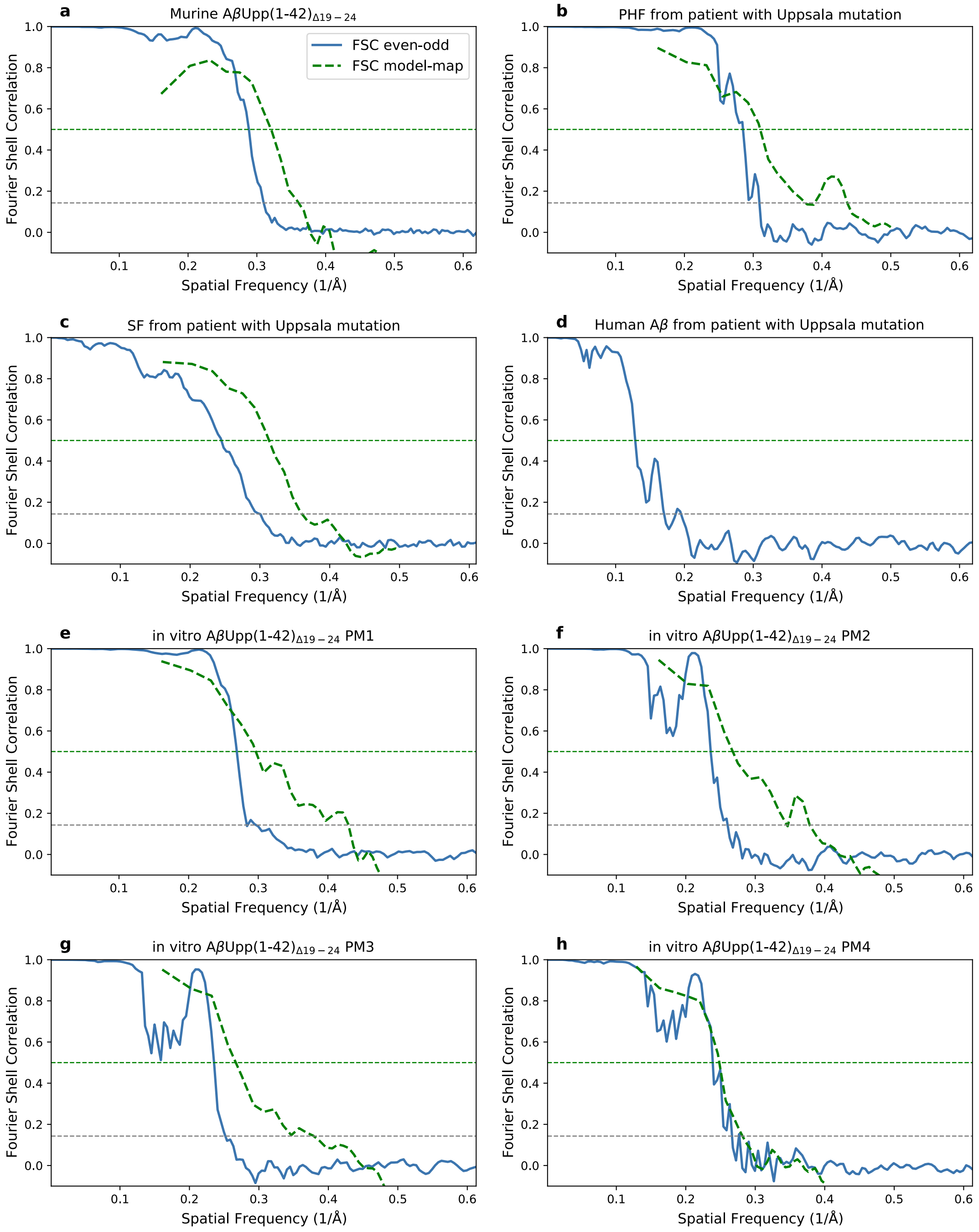

Figure S4: To demonstrate the similarity between the low-resolution AβUpp(1-42)$_{\Delta 19\text{-}24}$ reconstruction and the published density map (EMD-13800) of type I Aβ fibril from AD patient, the structure factor from the AβUpp(1-42)$_{\Delta 19\text{-}24}$ reconstruction was applied to the EMD-13800 density map, (**a**) shows both maps superimposed. (**b**) Showing atomic models after 100 ns of MD simulation for the WT Aβ(1-42) (brown) and AβUpp(1-42)$_{\Delta 19\text{-}24}$ (blue). The simulation of WT Aβ(1-42) was started from PDB ID 7Q4B and the simulation of AβUpp(1-42)$_{\Delta 19\text{-}24}$ a was started from a homology model of AβUpp(1-42)$_{\Delta 19\text{-}24}$ using PDB ID 7Q4B as a template. The red arrows indicate regions where the atomic model of AβUpp(1-42)$_{\Delta 19\text{-}24}$ deviates strongly from the density map. (**b**) Root-mean square deviation (RMSD) of the WT Aβ(1-42) (brown) and AβUpp(1-42)$_{\Delta 19\text{-}24}$ (blue) models from the respective starting models during the 100 ns simulations.

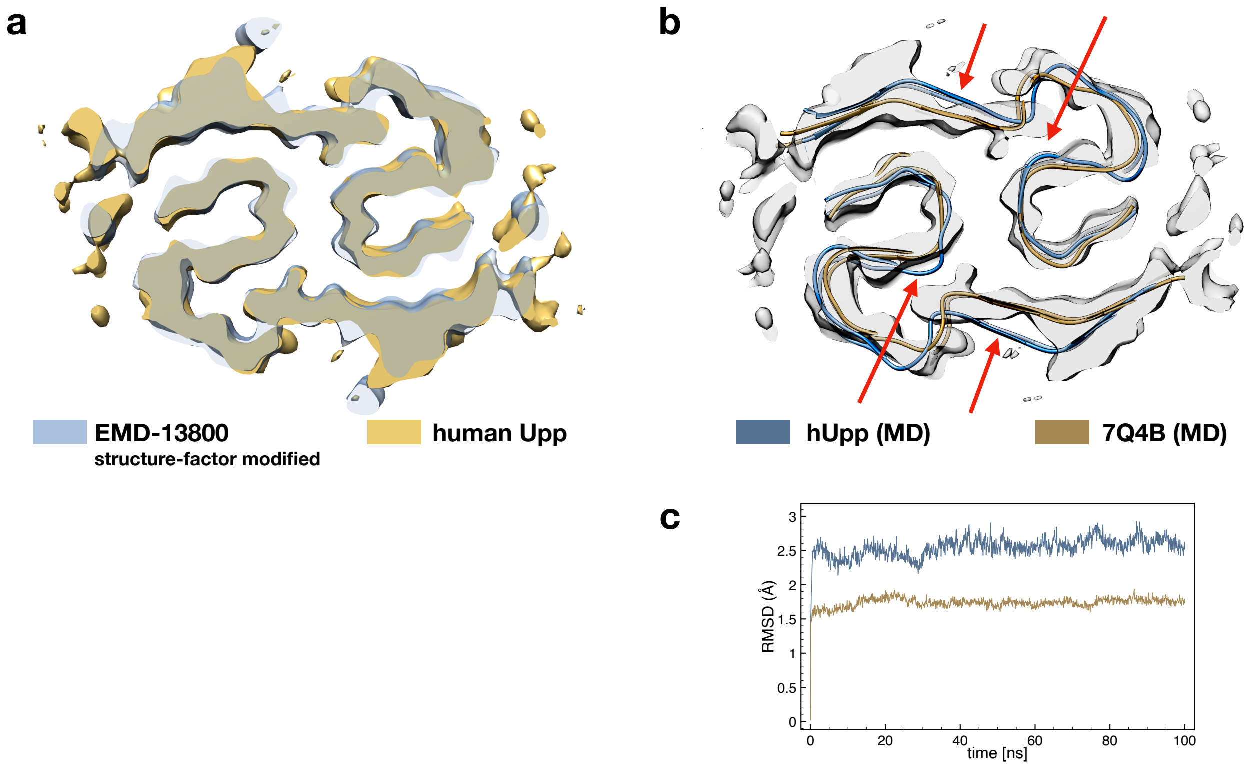

Figure S5: (**a**) Negative stain EM and (**b**) AFM images of *in vitro* AβUpp(1-42)$_{\Delta19\text{-}24}$ fibrils.

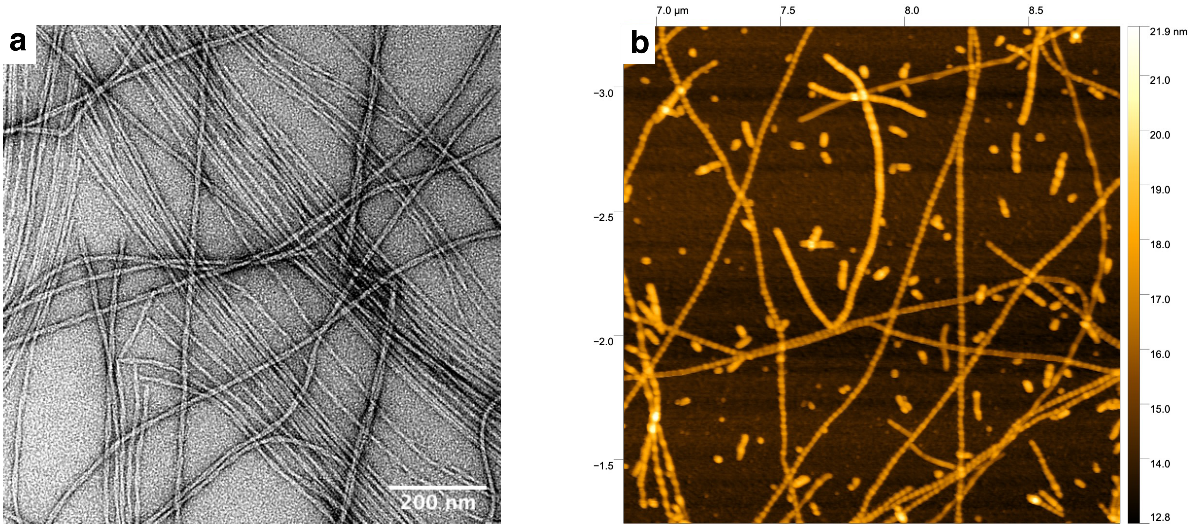

Figure S6: Determination of handedness of in-vitro polymorphs by comparison with high-resolution conserved C-terminal motif of PDB ID 7Q4B. For this, residues 30-40 (WT numbering) we fitted into the left- and right-handed density maps. In all four cases, the 7Q4B model clearly fits better to the left-handed fibril density than to the right-handed density, supporting a left-handed fibril in all cases.

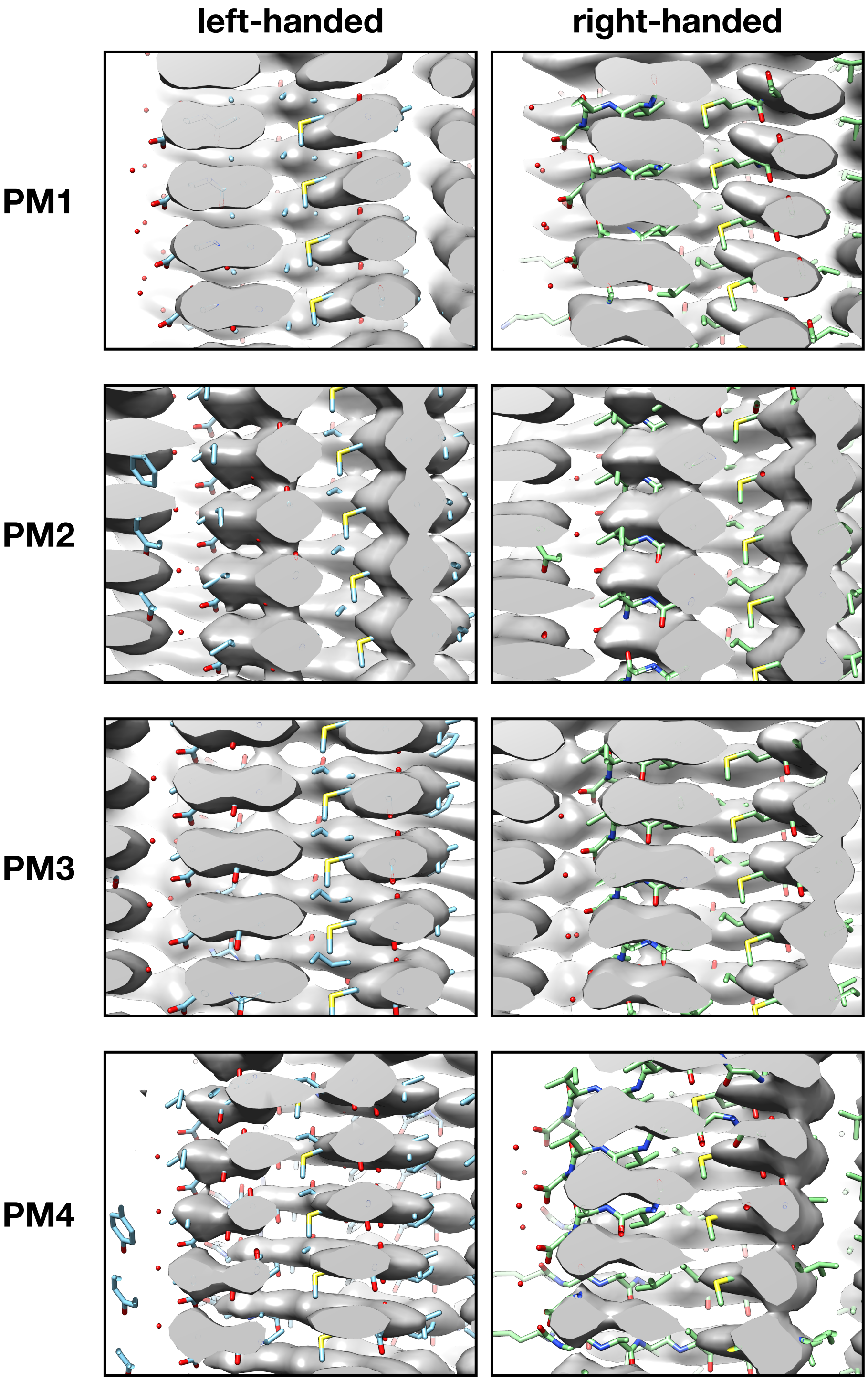

Figure S7: 2D class averages of fibrils processed from tg-UppSwe mouse brain tissue. These fibrils are all composed of AβUpp(1-42)$_{\Delta19\text{-}24}$. The class averages are taken from early steps of the classification and fibril selection process to provide an overview of the structural variability in the dataset.

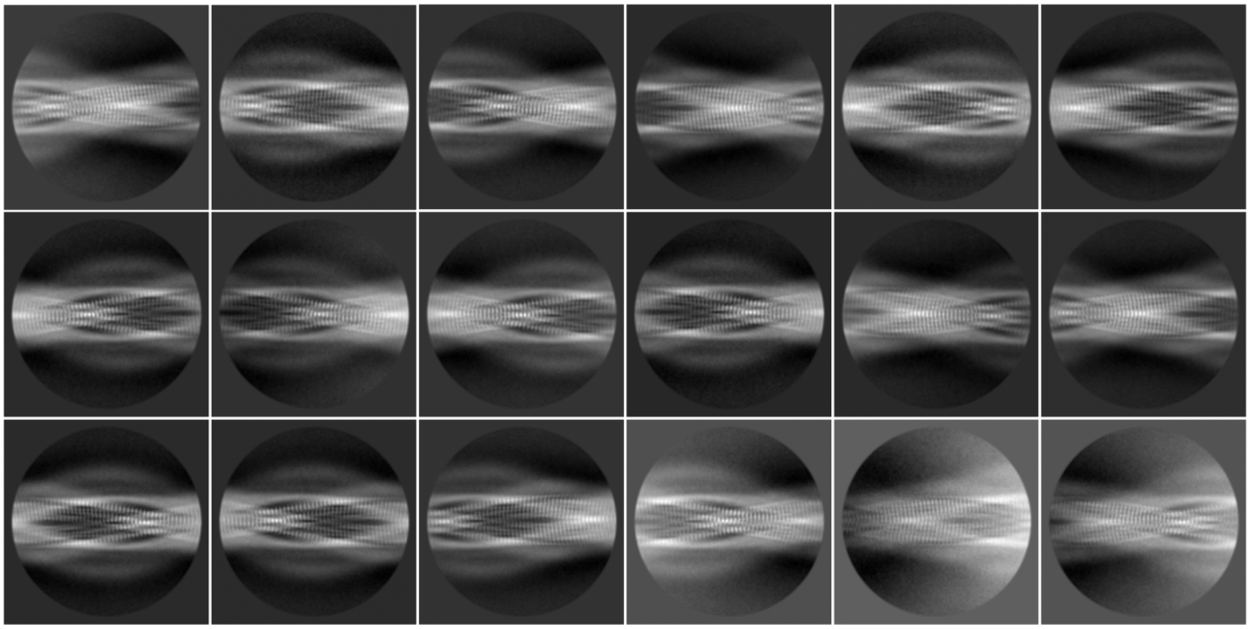

Figure S8: 2D class averages of fibrils processed from human brain tissue of an individual with the Uppsala mutation. (**a**) Dataset used for structure determination of the Aβ and PHF tau fibrils. (**b**) Dataset used for structure determination of the SF tau fibril. All class averages are taken from early steps of the classification and fibril selection process to provide an overview of the structural variability in the dataset.

a

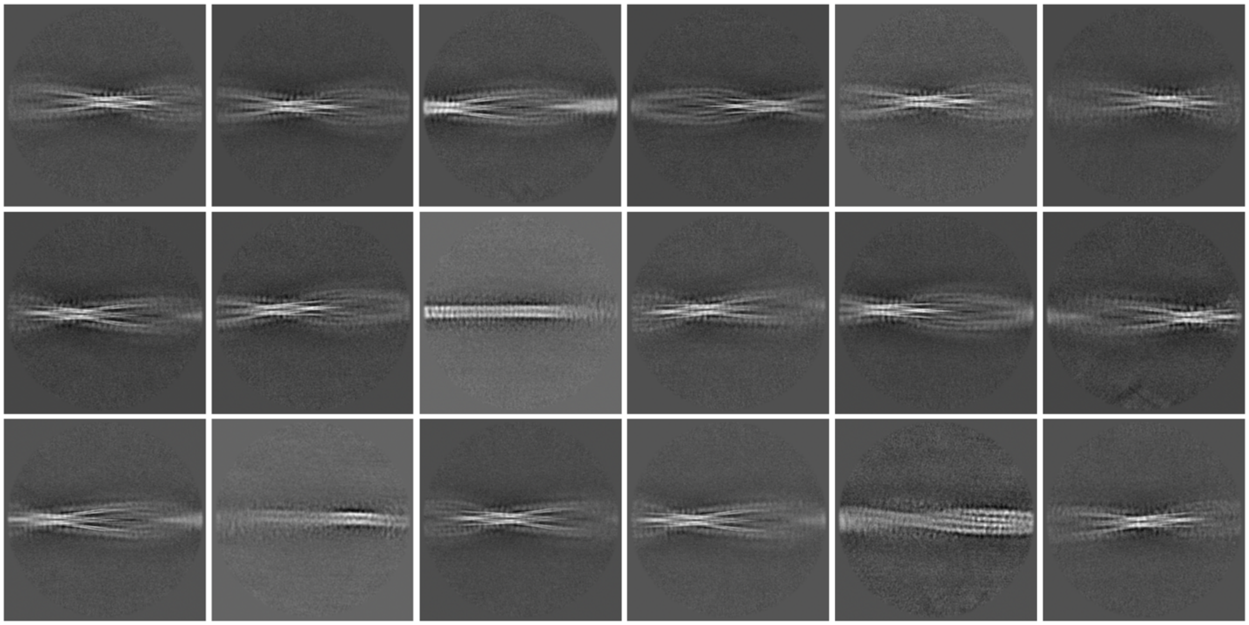

b

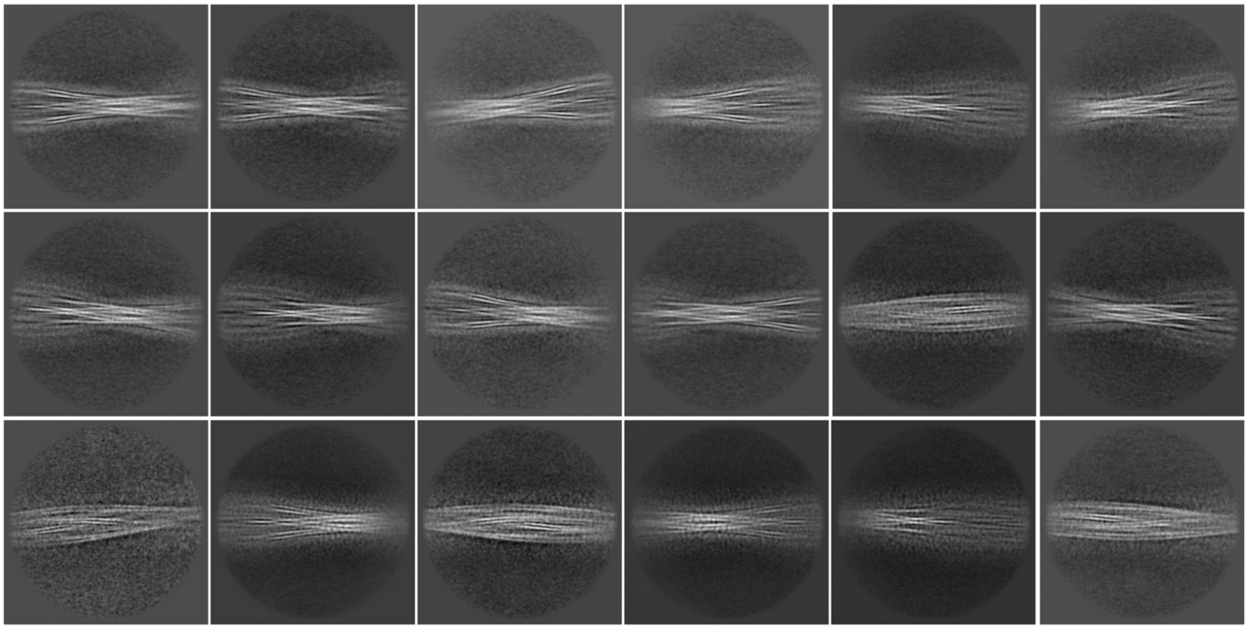

Supplement: Supplementary file 1 — Additional file 1: Supplementary Figures. [file 40478_2025_2120_MOESM1_ESM.pdf]
