# Supplementary material for: Cryo-EM studies of amyloid-β fibrils from human and murine brains carrying the Uppsala APP mutation (Δ690–695)
Source: Acta Neuropathol Commun. 2025 Oct 3;13:209. doi: 10.1186/s40478-025-02120-x (PMC12492897; doi:10.1186/s40478-025-02120-x)
Supplement: Supplementary file 2 — Additional file 2: Supplementary Table S1. [file 40478_2025_2120_MOESM2_ESM.pdf]

Supplementary Table 1: Cryo-EM data collection, refinement and validation statistics

	tg-UppSwe, AβUpp(1-42)$_{\Delta 19\text{-}24}$	Human Case, Tau SF	Human Case, Tau PHF	Human Case, Aβ	Synthetic AβUpp(1-42)$_{\Delta 19\text{-}24}$, PM1	Synthetic AβUpp(1-42)$_{\Delta 19\text{-}24}$, PM2	Synthetic AβUpp(1-42)$_{\Delta 19\text{-}24}$, PM3	Synthetic AβUpp(1-42)$_{\Delta 19\text{-}24}$, PM4
**Data collection and processing**								
Microscope	Titan Krios	Titan Krios	Titan Krios		Talos Arctica			
Detector	Falcon IV	K3	Falcon IV		K3			
Magnification	96,000	105,000	96,000		100,000			
Energy filter slit width (eV)	N/A	20	N/A		20			
Voltage (kV)	300	300	300		200			
Electron exposure (e^{-}/Å^{2})	40	58	40		50			
Defocus range (μm)	[-2.5,-0.5]	[-2.5,-0.75]	[-2.5,-0.75]		[-3.5,-1.5]			
Pixel size (Å)	0.808	0.82	0.808		0.816			
Micrographs collected	11,991	10,020	17,910		3,954			
Symmetry imposed	C1	C1	C1	C1	C1	C1	C1	C1
Helical rise (Å)	2.36	4.78	2.39	2.45	2.37	2.38	4.73	4.7
Helical twist (°)	178.4	-1.07	179.4	178.4	178.4	179.2	-2.2	-4.8
Initial particle images (no.)	1,271,423	2,303,266	515,788	515,788	1,104,683	429,320	366,776	214,319
Final particle images (no.)	329,437	19,195	112,719	5,023	155,170	66,738	52,721	133,988
Box size (pix)	300	300	270	300	270	270	270	270
Inter-box distance (pix)	14	14	14	14	17	17	17	17
Map resolution (Å)	3.2	3.4	3.3	5.9	3.4	3.9	4	3.8
FSC threshold	0.143	0.143	0.143	0.143	0.143	0.143	0.143	0.143
**Refinement**								
Initial model used (PDB code)	N/A	N/A	5O3L	N/A	N/A	N/A	N/A	N/A
Map sharpening *B* factor (Å^{2})	-134.6	-120.7	-105.3	N/A	N/A	N/A	N/A	N/A
Model composition		N/A		N/A				
Chains	10		10		10	10	10	20
Non-hydrogen atoms	2050		5570		1990	1990	1640	1740
Protein residues	290		730		280	280	240	280
R.m.s. deviations		N/A		N/A				
Bond lengths (Å)	0.01		0.01		0.01	0.01	0.01	0.01
Bond angles (°)	2.05		2.01		1.94	2.05	1.81	2.04
Validation		N/A		N/A				
MolProbity score	1.02		1.01		1.08	0.76	0.56	0.91
Clashscore	0.0		0		0	0	0	1.6
Ramachandran plot		N/A		N/A				
Favored (%)	99.6		91.3		88.9	96.2	97.7	100
Outliers (%)	0		0		0	0	0	0
